# Physical interpretation of Stephens’ phenomenological parameters for anisotropic microstrain broadening in cubic systems

**DOI:** 10.1107/S1600576726004176

**Published:** 2026-07-07

**Authors:** Ashok Bhakar, Pooja Gupta, Mahesh Kumar Swami, Sanjay Kumar Rai

**Affiliations:** a Indus Synchrotrons Utilization Division Raja Ramanna Centre for Advanced Technology Indore 452013 India; b Homi Bhabha National Institute Training School Complex Anushakti Nagar Mumbai 400094 India; Argonne National Laboratory, USA

**Keywords:** anisotropic peak broadening, microstrain, modified Williamson–Hall plots, Stephens’ phenomenological approach, whole powder pattern fitting, dislocation types, microstructure

## Abstract

Both the Stephens phenomenological model and the modified Williamson–Hall (mWH) plot analysis address the microstrain-dependent anisotropic peak broadening observed in diffraction patterns. The data analysis strategies of both methods rely on the anisotropic nature of crystalline materials and the quartic form of reflection indices (*hkl*). Despite these similarities, the results of the two methods are not directly comparable: the Stephens approach is phenomenological, whereas the mWH method is based on the characteristic dislocation parameters for determining microstructural features (size–strain). This study aims to bridge that gap. A simple procedure is proposed to extract microstructural quantities – equivalent to those obtained by the mWH method – using the Stephens phenomenological approach. Furthermore, a microscopic interpretation of Stephens’ phenomenological parameters is presented in terms of the nature of dislocations in cubic symmetry. The applicability of this procedure is demonstrated for cubic systems and illustrated with examples.

## Abstract 2

This study emphasizes the physical interpretation of Stephens’ phenomenological parameters with respect to the nature of dislocations in cubic symmetry, validated through illustrative examples based on modified Williamson–Hall plot analysis.

## 1. Introduction

Anisotropic peak profile broadening, *i.e.* when peak widths are not a smooth function of the scattering vector in powder diffraction patterns, is usually regarded as a hurdle in solving the crystallographic structure of materials. To address this, several approaches based on whole powder pattern fitting (WPPF) have been developed (Thompson, Reilly *et al.*, 1987 ▸; Rodriguez-Carvajal *et al.*, 1991 ▸; Popa, 1998 ▸; Stephens, 1999 ▸). In contrast, the same anisotropic nature of peak broadening is treated as a valuable source of information for microstructural characterization of materials (Snyder *et al.*, 1999 ▸; Mittemeijer & Scardi, 2004 ▸; Kaduk *et al.*, 2021 ▸). This latter approach is widely used in the materials science, metallurgy and engineering communities to determine nanocrystallite domains (shape, size and distribution) and crystalline defects (vacancies, dislocations, stacking, twin faults *etc*.). These methods are broadly classified as peak (or line) profile analysis (PPA or LPA) methods and also include more recently developed methods of whole powder pattern modeling (Warren & Averbach, 1950 ▸; Williamson & Hall, 1953 ▸; Ungár & Borbély, 1996 ▸; Scardi & Leoni, 2002 ▸; Scherrer, 1918 ▸; Ribárik *et al.*, 2020 ▸; Scardi, 2020 ▸; Materěj *et al.*, 2014 ▸).

The Stephens approach is widely employed in WPPF methods to account for anisotropic peak broadening caused by microstrain, without considering its origin (Stephens, 1999 ▸). Consequently, it is regarded as phenomenological in nature. In this framework, microstrain broadening is interpreted as a manifestation of the distribution of unit-cell parameters, *i.e.* each crystallite is imagined to have its own set of lattice parameters within the sample, and is modeled using a quartic form in reciprocal space. This description has been commonly adopted in the literature (Stokes & Wilson, 1944 ▸; Thompson, Reilly *et al.*, 1987 ▸; Rodriguez-Carvajal *et al.*, 1991 ▸; Sarkar *et al.*, 2007 ▸; Popa, 1998 ▸; Leineweber, 2011 ▸). This approach facilitates the calculation of accurate intensities in diffraction patterns exhibiting anisotropic peak broadening. Its incorporation is therefore essential for reliable crystal structure determination or for improving pattern decomposition outcomes in WPPF methods such as the Rietveld or Le Bail (structure-less) methods (Rietveld, 1967 ▸; Rietveld, 1969 ▸; Le Bail *et al.*, 1988 ▸). Despite their widespread use, the significance of Stephens’ phenomenological parameters (represented by *S_HKL_*) remains vague, and they are primarily applied in structural refinement (Dinnebier *et al.*, 1999 ▸; Stephens, 2019 ▸; https://www.researchgate.net/post/What_is_the_physical_significance_of_strain_S_factors_in_Stephens_broadening_model_of_microscopic_strain/1). Although attempts have been made to predict the physical meaning of *S_HKL_* since the method’s inception, these efforts have been ineffectively applied or extended to experimental datasets (Stephens, 2019 ▸). To the best of the authors’ knowledge, a comprehensive study on this matter has not yet been undertaken.

The modified Williamson–Hall (mWH) plot is another method for analyzing anisotropic peak broadening due to the microstrain effect (Ungár & Borbély, 1996 ▸). It is a modification of the Williamson–Hall (WH) plot, which represents the simplest case of isotropic peak broadening analysis (Williamson & Hall, 1953 ▸). In this approach, the concept of average contrast factor is employed to account for anisotropic peak broadening and dislocations are considered as the origin of the microstrain effect. Thus, the physical interpretation of the anisotropic nature of peak broadening is explicit in this case. This method focuses exclusively on the width part of diffraction peaks, without considering their intensity part, for microstructural (also known as size–strain) analysis. In doing so, it provides information complementary to structural/WPPF methods and is regarded as an appropriate approach for interpreting the meaning of *S_HKL_* parameters in this study.

Despite these differences, the two methods – the mWH plot and Stephens’ phenomenological model – share several similarities. First, they both address the microstrain-dependent anisotropic peak broadening contribution to diffraction patterns. The mathematical formalism and data analysis philosophy of the two methods are based on the quartic form of Bragg reflections (*hkl*) and correspond to the anisotropic nature of the elastic moduli of crystalline materials. Both approaches were developed around the same time, in the late 1990s (Ungár & Borbély, 1996 ▸; Stephens, 1999 ▸), and have been successfully applied to solve a wide range of problems over the past two and a half decades. Despite these similarities, the two approaches are rarely compared, and such a comparison is undertaken in this work. This study aims to illustrate the following:

(i) A detailed procedure for performing mWH plot analysis using the outcomes of WPPF methods that incorporate Stephens’ phenomenological approach for cubic systems.

(ii) The physical significance of *S_HKL_* parameters in assessing the character of dislocations.

This work focuses primarily on the anisotropic peak broadening contribution to diffraction patterns due to microstrain, with emphasis on cubic systems only. Its applicability is demonstrated through examples drawn from the literature.

## 2. Methods of analysis: mathematical details

A diffraction peak profile contains contributions from both instrumental and sample-related effects. In this work, the evolution of the peak profile is described only for sample effects, *i.e.* the instrumental part is either removed *a priori* or appropriately accounted for during the analysis. The physical contribution to peak profile broadening is the convolution of size–strain effects and simply expressed as
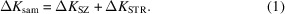
Here, the peak broadening parameter Δ*K* is related to the width (or breadth) of the diffraction peaks. The subscripts to Δ*K*, namely ‘SZ’ and ‘STR’, correspond to the crystallite size and microstrain (dislocations in particular, for this study) broadening contributions, respectively, to the total sample-related (sam) broadening.

In the literature, several empirical profile functions have been chosen (based on observations) to achieve better fitting of peak profiles, without consideration of their origin or relation to the microstructure of the samples. The pseudo-Voigt (pV) profile is among the most commonly used analytical functions for size–strain analysis. The pV profile is a linear combination of Lorentzian (L) and Gaussian (G) profiles of the same full width at half-maximum (FWHM), *i.e.* pV(*x*) = η L(*x*) + (1−η) G(*x*). In this section, the details of the mWH plot and Stephens’ phenomenological model are briefly compared to account for microstrain/dislocation-dependent anisotropic peak broadening. For PPA, either the FWHM or the integral breadth (IB, denoted as β) of diffraction peaks is used to characterize the peak broadening parameter Δ*K*. In this work, the IB-based approach is adopted for its direct comparability with the output of the WPPF method obtained using the *FullProf Suite* program (Rodríquez-Carvajal & Roisnel, 2004 ▸).

### 2.1. mWH analysis

Ungár & Borbély (1996 ▸) upgraded the classical WH plot by incorporating the contrast factors of dislocations to account for anisotropic (*hkl*-dependent) peak broadening. This method is popularly known as the mWH plot and is described as follows:

(i) Linear form:
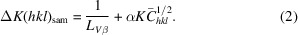


(ii) Quadratic form:
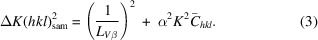
The scattering vector magnitude is *K* = 2 sin θ/λ. 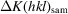 = 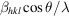, which is related to the *hkl*-dependent IB (β*_hkl_*) due to sample effects (after removing the instrumental contribution, *i.e.* the instrumental resolution function, IRF). θ corresponds to the scattering angles of various Bragg peaks and λ is the wavelength of the diffraction source.


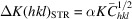
 represents the anisotropic IB due to microstrain, where α is the *hkl*-independent mean square strain. α corresponds to the slope of the mWH plot and is useful for extracting information about dislocations, as described in the literature (Ungár & Borbély, 1996 ▸; Borbély *et al.*, 2023 ▸). For this purpose, knowledge of the Wilkens arrangement parameter *M*, which depends on the effective outer cut-off radius of dislocations, is also essential (Wilkens, 1970 ▸; Wilkens, 1975 ▸; Borbély, 2022 ▸).


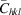
 is the *hkl*-dependent anisotropic average contrast factor due to dislocations, often simply denoted by 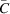. It depends on the elastic modulus of the crystal and the relative contributions of the edge and screw nature of dislocations.

For cubic systems, 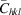 is expressed as
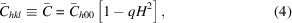
where *H*^2^ = (*h*^2^*k*^2^ + *k*^2^*l*^2^ + *l*^2^*h*^2^)/(*h*^2^ + *k*^2^ + *l*^2^)^2^ and *h*, *k*, *l* are indices of the diffraction peak. 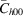 is the average contrast factor corresponding to *h*00 reflections. *q* is a fitting parameter that describes the edge/screw character of the dislocations.

Here, Δ*K*_SZ_ = 1/*L*_Vβ_ is taken as the *hkl*-independent IB due to the size effect in equations (2) and (3) as originally formulated by Ungár & Borbély (1996 ▸). It is calculated using the intercept of the mWH plot. *L*_*V*β_ represents the IB-based volume-average apparent crystallite size and the Scherrer constant is taken as unity (Scherrer, 1918 ▸; Langford & Wilson, 1978 ▸; Bhakar *et al.*, 2023 ▸). In fact, an anisotropic contribution (arising from different crystallite shapes) can also be considered in the analysis. However, since the aim of this study is to explore the microstrain contribution only (which is sufficient for physical interpretation of Stephens’ parameters), the size-effect part is kept to a bare minimum.

From mWH plot analysis, three physically relevant parameters can be obtained: (i) intercept (corresponding to the size contribution), (ii) slope (α) and (iii) *q*. In the following sections, the procedure for extracting two unknown microstrain-dependent parameters (α and *q*) using WPPF analysis is described.

### 2.2. WPPF analysis

In this study the procedure of WPPF analysis of diffraction patterns using the *FullProf* software (Rodriguez-Carvajal, 1993 ▸) and Thompson–Cox–Hastings (TCH) profile function (Thompson, Cox & Hastings, 1987 ▸) is described for the interpretation of anisotropic microstrain broadening obtained using Stephens’ phenomenological model. Further details are described in the literature (Stephens, 1999 ▸; Rodríquez-Carvajal & Roisnel, 2004 ▸). It is briefly outlined here for separating isotropic size broadening and anisotropic microstrain broadening contributions. The mathematical expressions of the TCH function for the Gaussian (*H*_G_) and Lorentzian (*H*_L_) components of the FWHM (*H*) of a diffraction pattern are given as (Rodríquez-Carvajal & Roisnel, 2004 ▸)
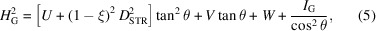

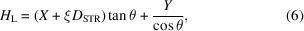

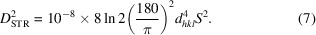
Here the parameters *U*, *V*, *W*, *I*_G_ have units of °^2^ while *X*, *Y*, *D*_STR_ are in ° and ξ is unit-less. *d_hkl_* is the interplanar spacing corresponding to the reflection indices *hkl* of the diffraction peak. Although TCH and pV functions are convertable to each other (Thompson, Cox & Hastings, 1987 ▸), in the *FullProf* program the TCH function is preferred for microstructural analysis; therefore, the same is adopted in this study. The convenience of using the TCH profile function lies in the fact that, when an IRF file is provided, the parameters that appear in equations (5) to (7) directly correspond to the intrinsic profile, *i.e.I*_G_ and *Y* (and *U* and *X*) are directly related to the Gaussian and Lorentzian contributions of isotropic size (and microstrain) broadening parts, respectively. *D*_STR_ describes anisotropic microstrain broadening and ξ is the phenomenological Lorentzian fraction (Stephens, 1999 ▸). The expression of *S*^2^ in terms of Stephens’ parameters *S_HKL_* (coefficients of the quadratic form of microstrain) is
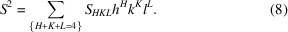


For cubic symmetry, refinement of only two independent *S_HKL_* parameters is required (namely *S*_400_ and *S*_220_) (Popa, 1998 ▸; Stephens, 1999 ▸; Leineweber, 2011 ▸); therefore, equation (8) simplifies as
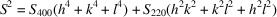
since 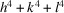 = (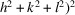 − 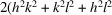). Therefore,
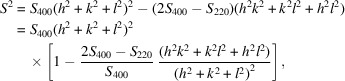

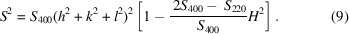


### 2.3. Procedure of mWH plot analysis using the parameters of the WPPF approach

The *FullProf* suite employs the concept of Stokes & Wilson (1944 ▸), along with the linear form of the classical WH plot, to generate a microstructural output file according to the following equation:
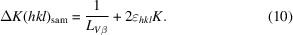


The anisotropic microstrain ɛ*_hkl_* is also referred to as maximum microstrain in the literature following Stokes & Wilson (1944 ▸) and is deduced from the Stephens’ *S_HKL_* parameters. The other terms of equation (10) are identical to those described in Section 2.1 above for the mWH plot. By equating the microstrain parts of the two methods, *i.e.* {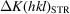} of the mWH plot = {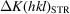} of WPPF, we obtain
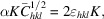
which leads to
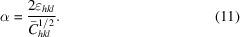


In this way, the slope-dependent parameter α can be determined from equation (11). Once α is known, it becomes possible to extract information equivalent to that obtained from mWH plot analysis, as described in the literature (Ungár & Borbély, 1996 ▸; Borbély *et al.*, 2023 ▸).

All anisotropic microstrain contributions are associated with the prefactor of the *H*^2^ term in equations (4) and (9), and are contained in 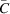 of the mWH method and *S*^2^ of the WPPF approach. Thus, the comparison of the terms enclosed in the square brackets of equations (4) and (9) yields the value of the *q* parameter as
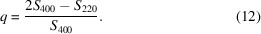
From this equation, the physical interpretation of Stephens’ phenomenological parameters becomes evident for cubic systems. Thus, *S_HKL_* parameters are directly associated with the character of dislocations.

In this work, the analysis procedure for the *FullProf* suite is described, but it is expected to be directly applicable to other WPPF programs that implement or follow the calculation of anisotropic *S*^2^ parameters as per equations (8) and (9). Different programs usually differ in their style of parameterization of peak broadening contribution through equations (5) to (9), in terms of their units such as degree, radian, centi-degree *etc*., and use of some prefactors or constant multipliers (Kaduk & Reid, 2011 ▸). Consequently, the values of *S_HKL_* parameters also differ depending on the mode of representation. However, equation (12) is independent of the units of the *S_HKL_* parameters as well as the use of any prefactor or multiplier, since it represents a ratio of quantities with the same dimensionality. Therefore, straightforward determination of the *q* parameter is possible across different WPPF-based programs.

## 3. Examples

The efficacy of the procedure outlined in Section 2 is now demonstrated by solving several examples from the literature. First, the approach is tested on a simple case of Fe powder, where peak broadening arises primarily from the microstrain effect. It is then applied to more complex situations in which both crystallite size and microstrain effects are present simultaneously.

### 3.1. Iron powder

Synchrotron X-ray diffraction (XRD) measurements of as-received Fe powder were carried out at the Engineering Applications Beamline (BL-02) of Indus-2, India (Gupta *et al.*, 2021 ▸). Super-Lorentzian peak shapes with anisotropic widths were observed and attributed to the presence of bimodal microstructures (Bhakar *et al.*, 2021 ▸). Instrumental correction was applied during the Rietveld refinement using an IRF file, and the bimodal profiles were deconvoluted into narrow and broad components. Output parameters of both profiles are provided in Table 1 ▸ for microstructural calculations. A detailed PPA of this powder, reported using the Rietveld method and the mWH plot approach, indicated that peak broadening is primarily due to the microstrain effect, with the crystallite size contribution being negligible. Thus, this dataset can be considered an ideal case for validating the proposed methodology and assessing its reliability for practical applications.

The calculated values of the α and *q* parameters [as per equations (11) and (12), respectively] are ≃ 1.17 × 10^−2^ and 2.11, respectively, for the broad profile and ≃ 3.4 × 10^−3^ and 2.2, respectively, for the narrow profile, as given in Table 1 ▸. These values are in excellent agreement with the slope α^*n*^ and *q* parameters of both profiles obtained using mWH plot analysis of the same datasets by Bhakar *et al.* (2021 ▸). The superscript *n* = 1 + √(1 − η)^2^ is related to the Lorentzian fraction (η) of the pV profile (Srikant *et al.*, 1997 ▸). Since *FullProf* employs the linear form (*i.e.n* = 1) of the WH plot, the values of *n* were 1.0225 and 1.28 for the narrow and broad microstructural profiles of Fe powder, respectively. Therefore, the slopes (α^*n*^) corresponding to the mWH plot are (3.4 × 10^−3^)^1.0225^ = 3 × 10^−3^ and (1.17 × 10^−2^)^1.28^ = 3.36 × 10^−3^ for the narrow and broad components of the Fe microstructure, respectively. These values are the same within the experimental uncertainties as in the mWH plots shown in Figs. 5(*b*) and 5(*c*) of Bhakar *et al.* (2021 ▸). This exact agreement confirms that mWH plot analysis is directly possible from the outcomes of WPPF methods employing Stephens’ phenomenological approach and the *S_HKL_* parameters are related to the *q* parameter in cubic systems. Further, mWH plots of the narrow and broad profiles were obtained using the linear [as per equation (2)] and quadratic form [as per equation (3)], yielding identical values of α and *q* parameters. This demonstrates that the α and *q* parameters are independent of peak shapes for this example of Fe powder, which is expected since both the widths and shapes of peak broadening are governed primarily by the microstrain effect.

### 3.2. Ball-milled Fe–Si–B powders

**Figure 1:**
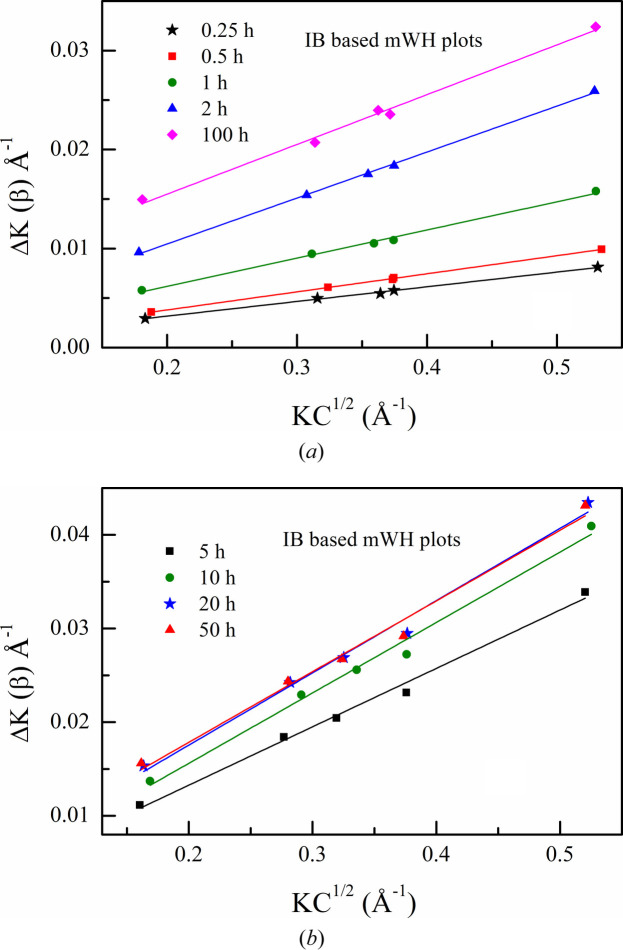
mWH plots corresponding to the b.c.c. phase of ball-milled Fe–Si–B powders for different milling times: (*a*) 0.25, 0.5. 1, 2 and 100 h; and (*b*) 5, 10, 20 and 50 h.

Ball milling was used to prepare Fe–Si–B (75:20:5 at.%) samples by mixing high-purity elemental powders of Fe, Si and B in their stoichiometric ratio. Further details are given elsewhere (Bhakar *et al.*, 2026 ▸). In this study, Fe–Si–B samples were prepared by varying the milling time from 0.25 to 160 h, while keeping all other milling conditions fixed. The laboratory XRD measurements revealed that the main phase of all samples corresponds to an α-iron-rich b.c.c. (body-centered cubic) Fe(Si,B) structure, with diffraction peaks exhibiting anisotropic broadening. Microstructural analyses of the XRD data of these ball-milled alloys were performed using the WPPF (Le Bail fitting) and mWH plot methods. Optimized output parameters (up to 100 h) are provided in Table 2 ▸ for the major Fe–Si–B phase, and the corresponding mWH plots are shown in Figs. 1 ▸(*a*) and 1 ▸(*b*). Initially, two-phase Le Bail fitting was required to account for an unmixed fraction of Si phase observed up to 2 h of ball milling. For longer milling times (5 to 100 h), single-phase fitting was sufficient.

**Figure 2:**
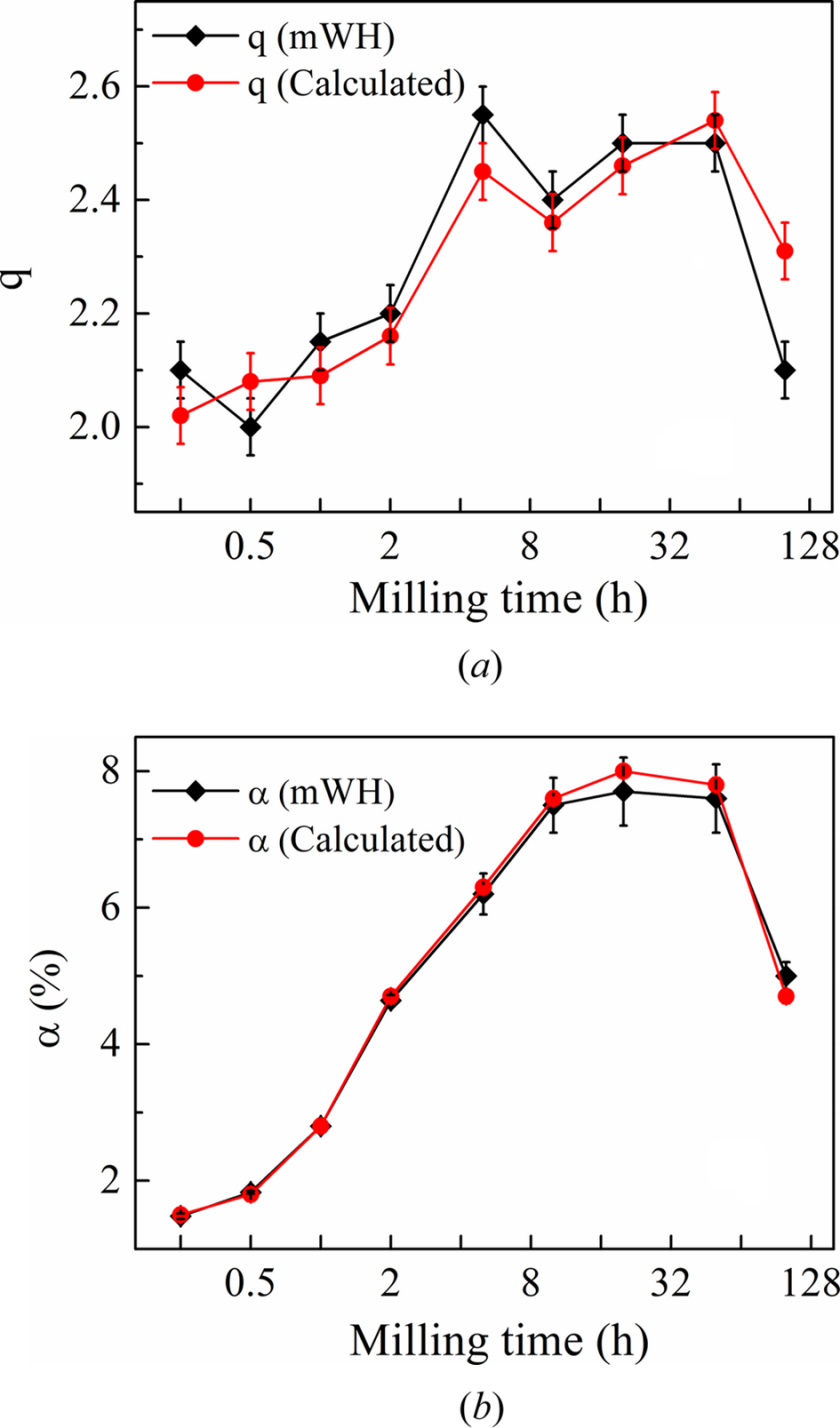
Comparison of (*a*) *q* and (*b*) α values obtained from mWH plot analyses and those calculated using the output parameters (*S_HKL_* are ɛ_*hkl*_) of WPPF with milling times.

Detailed PPA of Fe–Si–B samples suggests that both crystallite size and microstrain effects contribute to the peak broadening, with their relative contributions varying significantly with milling time (Bhakar *et al.*, 2026 ▸). This example can therefore be considered a representative case for validating the proposed methodology, comparable to the majority of practical situations. In this study, the linear form of the IB-based mWH plot is employed, enabling straightforward comparison of the α and *q* parameters with corresponding values calculated [as per equations (11) and (12)] using the output of the WPPF method. The *q* values obtained from the mWH plots are in good agreement with those calculated using the *S_HKL_* parameters in Table 2 ▸, lying in the range of 2 to 2.55, as compared in Fig. 2 ▸(*a*). Similarly, the α values estimated using the two methods are in excellent agreement for all nine samples, as presented in Fig. 2 ▸(*b*) and given in Table 2 ▸.

### 3.3. Rubidium fulleride, Rb_3_C_60_

Anisotropic peak broadening of the diffraction pattern of face-centered cubic (f.c.c.) Rb_3_C_60_ was modeled by Stephens (1999 ▸). The optimized values of the *S*_400_ and *S*_220_ parameters were 3.43 × 10^−8^ and −1.13 × 10^−8^, respectively. The calculated value of the *q* parameter is ≃ 2.33 using equation (12). It matches well with the experimentally determined *q* value of 2.47 (3) obtained from mWH plot analysis of the same diffraction data by Ungár *et al.* (1999 ▸). Calculation of the α parameter is not possible in this case, as ɛ_*hkl*_ values are not reported.

### 3.4. Ni_0.9_Zn_0.1_O

Rietveld refinement of the f.c.c. phase of various Ni_0.9_Zn_0.1_O samples was carried by Kremenović *et al.* (2010 ▸) using the *FullProf* software, with anisotropic peak broadening modeled using the Stephens approach. The refined values of the *S*_400_ and *S*_220_ parameters were 16.7 (7) and 39 (2) for the as-received sample; 16 (1) and 21 (2) for the sample annealed at 773 K for 3 h; 15 (1) and 20 (2) for the sample annealed at 773 K for 24 h; and 5.0 (6) and 7 (1) for the sample annealed at 973 K for 3 h, respectively. Correspondingly the calculated *q* parameter is ≃ −0.34 for the as-received sample and lies in the range ≃ 0.6–0.7 for all three annealed samples, as determined using equation (12). These *q* values indicate the presence of predominantly edge dislocations. In order to find the *q* values for the edge (*q*_E_) and screw (*q*_S_) character of the dislocation, the *ANIZC* program was used (Borbély *et al.*, 2003 ▸). The elastic moduli of NiO are considered (as elastic moduli for Ni_0.9_Zn_0.1_O are not available in the literature). This yielded *q*_E_ ≃ 0.65–0.9 and *q*_S_ ≃ 1.6–1.85 for the reported ranges of elastic constants for NiO (C_11_ = 271 – 270 – 224 GPa, C_12_ = 125 – 140 – 97 GPa and C_44_ = 105 – 95 – 110 GPa) (Liu *et al.*, 2008 ▸; Bartel & Morosin, 1971 ▸; Uchida & Saito, 1972 ▸; Towler *et al.*, 1994 ▸). Using the advanced tool of whole powder pattern modeling, Kremenović *et al.* also concluded that only edge dislocations were refinable in these samples, in agreement with the predictions of the present work.

These examples clearly demonstrate the utility of the proposed methodology for cubic systems with fairly complex microstructures. Although both methods (mWH and Stephens approach) are widely used in the literature, they are typically applied independently, which means verification was only possible in cases where both methods were employed simultaneously. In some reports, moreover, not all parameters required for calculation or validations are provided. XRD analyses of solution-annealed and isothermally aged 17-4 PH stainless steel using the Stephens approach and the *BRASS* code (https://www.brass.uni-bremen.de/) were reported by Manoj Kumar (2020 ▸). The refined values of the *S*_400_ parameter range between 12.75 × 10^−2^ and 8.20 × 10^−2^, while *S*_220_ ranges between −3.71 × 10^−4^ and −1.30 × 10^−2^ for the b.c.c. phase of different samples [from Table 4.2 of Manoj Kumar (2020 ▸)]. The *q* values calculated using equation (12) fall within the range of 2.1–2.3 for various samples. However, the corresponding *q* values were not reported, and the details of instrumental correction remain unclear. Therefore, a legitimate comparison is not possible. Still, by equating with *q* values of pure Fe, the dominance of the screw type of dislocations is expected in all 17-4 PH samples. We emphasize that reliable estimation of the *q* parameter is achieved using equation (12), despite the very different relative values of *S_HKL_* (spanning several orders of magnitude) obtained using different software packages [*GSAS* (Toby & Von Dreele, 2013 ▸), *FullProf*, *BRASS*]. These differences arise from the specific modes of parameterization employed, as seen from the analyzed examples in this section.

## 4. Discussion

One of the initial estimations of the *q* parameter using *S_HKL_* parameters was attempted by Ungar & Tichy (2002 ▸). They considered *q* as a simple ratio of *S*_400_/*S*_220_ and applied it to the cubic Rb_3_C_60_ system, which yielded *q* = −3.035. However, their experimentally determined value was 2.47 (3) (Ungár *et al.*, 1999 ▸), showing a significant deviation. The *q* value obtained using equation (12) is 2.33, which compares well with the experimental value and validates its appropriateness.

Direct application of this procedure for calculating the α and *q* parameters using the TCH profile and the output generated from the *FullProf* software has been demonstrated for several experimental datasets in Section 3 above. The results obtained by using the Stephens parameters from WPPF analysis of cubic systems are in excellent agreement with the mWH plot. This naturally raises the question: why adopt this new approach when the results are equivalent to the existing method of mWH plot analysis? The following benefits of the WPPF-based methodology help to answer this:

(i) Simultaneous determination of structural and microstructural parameters is possible using the Rietveld method, whereas the mWH plot provides only microstructural parameters. This dual capability helps establish more appropriate structure–property correlations.

(ii) Pattern decomposition in WPPF methods enables easier analysis and quantification of overlapping patterns such as double-indexed peaks (*e.g.* 330–411 of Fe), bimodal or multiple phases, and lower-symmetry crystals. Representative examples of severe peak overlap (cubic systems) where Rietveld refinement was essential include the bimodal study of Fe powder (Bhakar *et al.*, 2021 ▸) and Si powder (Bhakar *et al.*, 2024 ▸) and multi-phase analysis of lead magnesium niobate (Bhakar *et al.*, 2016 ▸; Bhakar *et al.*, 2017 ▸). In these cases, examining the quality of WPPF fits to diffraction peaks was an effective way of identifying bimodal microstructures (responsible for complex peak shapes), which are usually inaccessible through WH or mWH plot analysis.

(iii) Separation of peak shape contributions from size and strain effects is directly possible with WPPF methods as per Section 2 above. The importance of peak shape analysis lies in the fact that the Wilkens arrangement parameter *M* can vary by up to an order of magnitude when microstrain-dependent peak shapes shift from Gaussian-like to Lorentzian-like (Borbély, 2022 ▸). This strongly affects the reliable estimation of dislocation density. Likewise, peak shape information arising solely from the size effect is valuable for determining the crystallite size distribution (Bhakar *et al.*, 2023 ▸).

Conversely, prior knowledge of *q* values is also helpful for refining *S_HKL_* parameters when they diverge during WPPF fitting. For example, in the case of α-Fe, the *q* values are *q*_E_ = 1.3 (edge) and *q*_S_ = 2.7 (screw), representing two extreme cases of the character of dislocations. Using equation (12), it is possible to constrain the *S*_220_ values according to the following relationship:
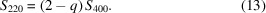
Thus, the upper and lower bound on *S*_220_ can be fixed at ±0.7*S*_400_ when fitting diffraction data for the Fe sample. In this case, positive, negative and zero values for the *S*_220_ parameter are possible. Similarly, for Si powder, only positive values of the *S*_220_ parameter are possible according to equation (13), because *q*_E_ = 0.75 and *q*_S_ = 1.73 for pure Si. This argument is also valid for other cubic systems with *q* values (*q*_E_ and *q*_S_) less than 2, such as the f.c.c. phase of Ni_0.9_Zn_0.1_O samples, for which the refined values of both *S*_400_ and *S*_220_ parameters are positive only, as reported by Kremenović *et al.* (2010 ▸).

In WPPF methods, it is crucial to provide a good starting model and to optimize the refinement sequence of parameters. Several strategies for sequential or simultaneous refinement of parameters are advised in the literature. A systematic, step-by-step parameter turn-on sequence is usually the most effective way to achieve convergence (Young, 1993 ▸; McCusker *et al.*, 1999 ▸; Toby, 2006 ▸). This approach is applicable to the analysis of Fe as well, *i.e.* optimizing *S*_400_ (corresponds to *q* = 2) in the earlier steps and then refining *S*_220_ in the next step. However, in the case of Si, simultaneous turn-on of both *S*_400_ and *S*_220_ parameters is more effective than refining only *S*_400_. This is because refining only *S*_400_ leads to an unrealistically large *q* value of 2 and increases the chances of divergence.

In this way, prior information about the actual or even approximate values of the *q* (*q*_E_ and *q*_S_) parameters is useful for deciding the appropriate refinement sequence for a particular material. Thus, both methods (WPPF and mWH plot) benefit mutually from the proposed strategy of this work, which provides a physical interpretation of phenomenological *S_HKL_* parameters in terms of *q* values for cubic systems. This approach can also be extended to other crystal systems for broader applicability.

## 5. Conclusions

The physical interpretation of the Stephens phenomenological parameters is presented in terms of the nature of dislocations for cubic symmetry. In this context, a mathematical relationship between the Stephens phenomenological parameters *S_HKL_* and the physical quantity *q*, which describes the character of dislocations, is derived. Additionally, a method for calculating the *hkl*-independent microstrain parameter α from the outputs of WPPF approaches is outlined. This enables a straightforward procedure for mWH plot analysis, using the parameters of Stephens’ phenomenological approach derived from WPPF methods (such as Rietveld and Le Bail) for cubic symmetry.

The practical applicability of the proposed procedure has been demonstrated using examples from the literature. In these cases, microstructural parameters were quantified to yield outcomes (*q* and α) equivalent to those obtained from mWH plot analysis. It is hoped that this work will stimulate further studies in this direction and that the methodology will be extended to other crystal systems for generalized use.

## Figures and Tables

**Table 1:** Calculated values of microstrain parameter α from the individual reflections of the broad and narrow profiles of Fe powder, obtained using the output parameters [*K*, Δ*K*(*hkl*)_sam_, *q* and ɛ_*hkl*_] of the WPPF (Rietveld) method and equation (11) Here 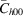 = 0.285. The *q* values are estimated using the *S_HKL_* parameters and equation (12). Numbers in parentheses indicate the estimated standard uncertainty (e.s.u.) in the last decimal place, while numbers without parentheses are either constant or rounded-off such that the e.s.u. lies within the last decimal place.

			Broad profile			Narrow profile		
*K* (Å^−1^)	*hkl*	*H* ^2^	Δ*K*(*hkl*)_sam_ (10^−3^ Å^−1^)	ɛ_*hkl*_ (10^−4^)	α (10^−2^)	Δ*K*(*hkl*)_sam_ (10^−3^ Å^−1^)	ɛ_*hkl*_ (10^−4^)	α (10^−3^)
0.4933	110	0.25	2.11	21.4	1.17	0.6	6.1	3.4
0.6976	200	0	4.34	31.1	1.17	1.27	9.0	3.4
0.8544	211	0.25	3.66	21.4	1.17	1.05	6.1	3.4
0.9866	220	0.25	4.22	21.4	1.17	1.2	6.1	3.4
1.1031	310	0.09	6.18	28.0	1.17	1.8	8.1	3.4
1.2083	222	0.33333	4.09	17.0	1.17	1.14	4.7	3.4
1.3052	321	0.25	5.58	21.4	1.17	1.6	6.1	3.4
1.3953	400	0	8.69	31.1	1.17	2.55	9.1	3.4
1.4799	330	0.25	6.33	21.4	1.17	1.8	6.1	3.4
1.4799	411	0.10185	8.16	27.5	1.17	2.38	8.0	3.4
1.56	420	0.16	7.9	25.3	1.17	2.3	7.3	3.4
*S*_400_, *S*_220_ and *q* (calculated)			35.6 (4), −3.8 (8) and 2.11 (20)			2.52 (6), −0.51 (9) and 2.2 (2)		

**Table 2:** Comparison of the microstrain parameters (α and *q*) of Fe–Si–B powders obtained using the WPPF and mWH plot approaches is presented for different milling times All essential parameters derived from the Le Bail fitting of the b.c.c. Fe–Si–B phase are reported in the *FullProf* format for calculating α and *q* values using 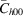 = 0.285. Numbers in parentheses indicate the e.s.u. in the last decimal place/s. The e.s.u. values for 110/210/220 and 310 reflections are smaller than those of the 200 reflection for the ɛ_*hkl*_ parameter; therefore, they are omitted.

	mWH results		WPPF output parameters						
						ɛ_*hkl*_ (10^−4^)			
Time (h)	α (%)	*q* (±0.05)	*S*_400_	*S*_220_	*q* (±0.05)	200	110/210/220	310	α (±0.1) (%)
0.25	1.48 (4)	2.1	52 (1)	−1 (2)	2.02	40 (1)	28	36	1.5
0.5	1.83 (2)	2.0	67 (2)	−6 (3)	2.08	49 (1)	34	44	1.8
1	2.8 (1)	2.15	43 (4)	−4 (2)	2.09	75 (4)	52	68	2.8
2	4.64 (8)	2.2	31 (2)	−5 (2)	2.16	125 (25)	85	112	4.7
5	6.2 (3)	2.55	950 (20)	−430 (35)	2.45	170 (6)	105	150	6.3
10	7.5 (4)	2.4	1335 (35)	−480 (65)	2.36	200 (9)	130	80	7.6
20	7.7 (5)	2.5	1525 (40)	−705 (73)	2.46	214 (10)	133	190	8.0
50	7.6 (5)	2.5	1450 (60)	−790 (105)	2.54	207 (15)	125	180	7.8
100	5.0 (2)	2.1	550 (30)	−170 (60)	2.31	125 (12)	82	112	4.7
